# RSPO1/β-Catenin Signaling Pathway Regulates Oogonia Differentiation and Entry into Meiosis in the Mouse Fetal Ovary

**DOI:** 10.1371/journal.pone.0025641

**Published:** 2011-10-03

**Authors:** Anne-Amandine Chassot, Elodie P. Gregoire, Rowena Lavery, Makoto M. Taketo, Dirk G. de Rooij, Ian R. Adams, Marie-Christine Chaboissier

**Affiliations:** 1 INSERM, U636, Nice, France; 2 Université de Nice-Sophia Antipolis, Laboratoire de Génétique du Développement Normal et Pathologique, Nice, France; 3 Department of Pharmacology, Graduate School of Medicine, Kyoto University Yoshida-Konoé-cho, Sakyo, Kyoto, Japan; 4 Center for Reproductive Medicine, Academic Medical Center, Amsterdam, The Netherlands; 5 Department of Endocrinology and Metabolism, Faculty of Science, Utrecht University, Utrecht, The Netherlands; 6 MRC Human Genetics Unit, Institute of Genetics and Molecular Medicine, Western General Hospital, Edinburgh, Scotland; Institut Jacques Monod, France

## Abstract

Differentiation of germ cells into male gonocytes or female oocytes is a central event in sexual reproduction. Proliferation and differentiation of fetal germ cells depend on the sex of the embryo. In male mouse embryos, germ cell proliferation is regulated by the RNA helicase *Mouse Vasa homolog* gene and factors synthesized by the somatic Sertoli cells promote gonocyte differentiation. In the female, ovarian differentiation requires activation of the WNT/**β**-catenin signaling pathway in the somatic cells by the secreted protein RSPO1. Using mouse models, we now show that *Rspo1* also activates the WNT/β-catenin signaling pathway in germ cells. In XX *Rspo1^−/−^* gonads, germ cell proliferation, expression of the early meiotic marker *Stra8*, and entry into meiosis are all impaired. In these gonads, impaired entry into meiosis and germ cell sex reversal occur prior to detectable Sertoli cell differentiation, suggesting that β-catenin signaling acts within the germ cells to promote oogonial differentiation and entry into meiosis. Our results demonstrate that RSPO1/β-catenin signaling is involved in meiosis in fetal germ cells and contributes to the cellular decision of germ cells to differentiate into oocyte or sperm.

## Introduction

Germ cells have the unique capacity to ensure the propagation of genetic information between generations. Once they are sex determined, they become competent for sexual reproduction by undergoing meiosis [1], [2]. During fertilization, male and female gametes join to form a diploid zygote with a mix of maternal and paternal heritable information. The sex of the resulting individual is determined by paternal transmission of either the Y chromosome that triggers testis development (XY), or the X chromosome, which promotes the formation of an ovary [3].

Male gonad development starts with somatic cell differentiation that is initiated by expression of *Sry*, an activator of the transcription factor *Sox9* [4]. In turn, activation of SOX9 leads to differentiation of somatic cells into Sertoli cells [5]. *Rspo1* and *Wnt4* are required for ovarian somatic differentiation [6], [7], [8], [9]. Loss-of-function of either *Rspo1* or *Wnt4* promotes sex reversal of the supporting cell lineages in XX gonads with differentiation of Sertoli cells around birth and development of ovotestes. RSPO1 is synthesized and secreted by somatic cells. RSPO proteins are regulators of the canonical WNT/**β**-catenin signaling pathway [10] and *in vitro* mediate their action through LRP6, the co-receptor of this signalling pathway [11], [12], [13]. However, the molecular mechanism remains to be elucidated. *In vitro*, RSPO1 can compete with DKK1, a negative regulator of the WNT/**β**-catenin signalling pathway, by binding to Kremen1 and then triggering the release of LRP6 [14]. However, Kremen receptors do not seem to be crucial for *Rspo1* signaling *in vivo* [15]. It has been shown that RSPO1 binds directly to LRP6 [11], [12] suggesting that this interaction is involved in transduction of the signal. This signal promotes stabilization of β-catenin which can then interact with the transcription factors LEF/TCF to induce expression of downstream target genes [16]. In ovaries, RSPO1 activates the β-catenin signaling pathway, promoting the up-regulation of *Wnt4* and differentiation of follicular cells [6].

In both XX and XY embryos, primordial germ cells migrate through the hindgut to colonize the gonad at around E10.5 [17]. In XY, but not XX fetal gonads, *Mvh* (*Mouse vasa homolog*) is required for germ cell proliferation [18] indicating that fetal germ cell proliferation is regulated by different pathways in XX and XY fetal gonads. However, the pathway(s) inducing germ cell proliferation in fetal ovaries remain to be elucidated. In fetal ovaries, germ cells proliferate, differentiate into oogonia and enter meiosis at E12.5–13.5. In the fetal testis, germ cells differentiate into gonocytes and become blocked in the G0/G1 phase of the cell cycle at around E14.5 [19]. Proliferation of these cells will only resume after birth when spermatogenesis begins. Thus, the initial decision to become an oocyte or a gonocyte is closely coupled with differential regulation of the cell cycle of fetal germ cells [20].

In XX fetal gonads, it has been shown that the onset of meiosis requires up-regulation of the *Stra8* gene in fetal oogonia [21]. Retinoic acid (RA) can induce *Stra8* expression in mouse fetal germ cells in organ cultures [22], [23], [24]. *Stra8*-deficient germ cells normally proliferate, but they fail to undergo premeiotic DNA replication and meiosis in fetal ovaries and arrest as premeiotic germ cells [21]. In rats that are nutritionally deficient for vitamin A, foetal XX germ cells have defects in upregulation of *Stra8* and in meiosis [24]. However, it has been reported that in XX *Raldh2^−/−^* embryos, *Stra8* expression is up-regulated normally in fetal oogonia in the absence of physiologically detectable RA levels *in vivo* [25]. Thus multiple signaling pathways may be involved in *Stra8* induction and meiosis initiation in mammalian gonads. In addition, RA promotes germ cell proliferation and germ cell survival in cultured embryonic ovaries [26], [27] underlying the multiple roles of RA in germ cell fate in embryonic ovaries.

In XY gonads, *Cyp26b1*, which is a member of a family of enzymes that degrades RA, is required to prevent *Stra8* expression in mouse fetal testes *in vivo* and *in vitro* [28], [29]. Tight control of RA levels is important for testicular development since RA can impair peritubular myoid cell migration and affect Sertoli cell differentiation in cultured rat embryonic testes [30]. In the developing testis, Sertoli cells, the somatic cells required to support spermatogenesis, contribute to gonocyte differentiation [31]. Consequently, defects in Sertoli cell differentiation promote male-to-female sex reversal of germ cells [5]. Indeed, XY germ cell sex reversal can be achieved by blocking the secretory pathway in cultured fetal testes [32], indicating that secreted factors, presumably originating from Sertoli cells, are required for male germ cell differentiation. Sertoli cells and *Sox9* are required for *Fgf9* up-regulation in the fetal testis [33], [34]. FGF9 is a secreted growth factor that promotes Sertoli cell differentiation and proliferation [34] and inhibits germ cell meiosis in culture [35], [36]. In addition to environmental signals, germ cell meiosis is also controlled by intrinsic factors that favor or prevent meiosis during embryogenesis [37], [38]. Indeed, the translational regulator *Nanos2* is required to maintain germ cells in G0/G1 phase in the fetal testis and ectopic expression of this gene in XX germ cells prevents entry into meiosis [39].

It is now clear that somatic and germ cell factors are required for oogonia to enter meiosis. Here we show that *Rspo1* directly activates β-catenin in XX germ cells. In turn, RSPO1/β-catenin signaling promotes XX germ cell proliferation and entry into meiosis.

## Results

### RSPO1 promotes XX germ cell proliferation

**Figure 1:**
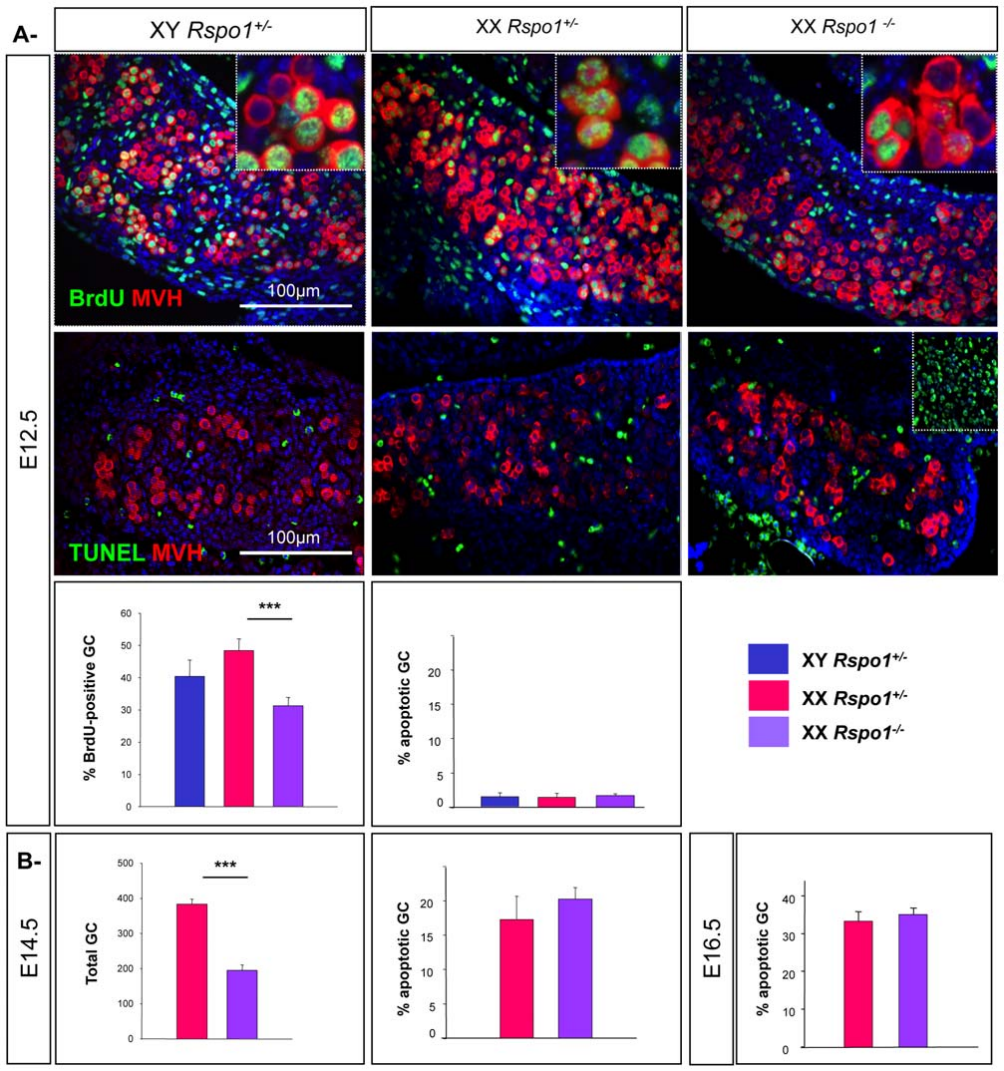
*Rspo1* promotes XX germ cells proliferation. A- Reduction of germ cell proliferation in XX *Rspo1* mutant gonads. Immunodetection of the proliferating germ cells with BrdU and MVH (upper panel), and the apoptotic germ cells with TUNEL and MVH (middle panel), in XY and XX *Rspo^+/−^*, and XX *Rspo1^−/−^* gonads at E12.5 gonads. DAPI (blue): nuclei. Inset middle panel: positive control for TUNEL. Histograms: Percentage of proliferating germ cells or apoptotic germ cells in XY, XX *Rspo1^+/−^* and *Rspo1^−/−^* gonads at E12.5. Bars represent mean+1 SEM, n = 24 sections of each genotype. B- Ablation of *Rspo1* does not trigger germ cell apopotosis. Quantification of germ cell number and germ cell apoptosis in XX *Rspo1^+/−^* and *Rspo1^−/−^* gonads at E14.5 and E16.5. Bars represent mean+1 SEM, n = 24 sections of each genotype.

Although the precise mechanisms controlling germ cell proliferation in XY fetal gonads remain to be clarified, they involve the RNA helicase protein MVH [18]. In XX gonads, MVH is expressed in the germ cells but is not required for oogonial proliferation indicating that the regulation of the proliferation of female fetal germ cells involves distinct molecular pathways. RSPO1 has been shown to regulate proliferation [40]. This signaling protein was found bound to the cellular membrane of the germ cells and somatic cells of the ovaries [10], [41], [42] suggesting that RSPO1 plays a role not only in somatic but also in germ cell proliferation. In addition, *Rspo1* becomes strongly up-regulated in the somatic cells of the XX gonad from E11.5 onwards [6], [7], [43] when germ cells are highly proliferative. To address whether this secreted protein might promote germ cell proliferation in XX gonads, we carried out bromodeoxyuridine (BrdU) labeling experiments at E12.5 in XX *Rspo1* mutant and control gonads (Fig. 1A). In XX *Rspo1^−/−^* gonads and in XY and XX controls, BrdU positive cells were detected, implying that germ cells were proliferating. In XX *Rspo1^−/−^* gonads, the percentage of germ cells that incorporated BrdU was significantly reduced compared to XX controls (31% in XX *Rspo1^−/−^* gonads versus 48% in XX controls), suggesting a decrease in germ cell proliferation. However, some reduction of BrdU incorporation could also be due to pre-meiotic S phase failure in germ cells within the mutant gonads. As premeiotic S phase is not followed by cell division, we were able to assess the relationship of this reduction in BrdU incorporation to germ cell proliferation, by counting the number of germ cells at E14.5 in XX controls and XX *Rspo1^−/−^* gonads. Mutant mice showed a small, but statistically significant decrease of germ cells at this stage (Fig. 1B).

*Rspo1* is involved in *Wnt4* up-regulation in XX gonads. Indeed, *Wnt4* is expressed in XX and XY gonads at a basal level [33] and *Rspo1* is required for the up-regulation of *Wnt4* in the XX gonad. Previous results showed that 90% of germ cells undergo apoptosis in XX *Wnt4^−/−^* gonads [8]. In addition, analysis of another designed *Rspo1* mutant showed an increase of cell apoptosis potentially germ cells in XX *Rspo1* mutant gonads at E16.5 [43]. However, statistical analysis showed no significant increase in germ cell apoptosis (TUNEL experiments) takes place at E12.5, E14.5 or at E16.5 in XX *Rspo1^−/−^* gonads when compared to XX controls (Fig. 1). This suggests that some remaining *Wnt4* expression can still promote survival of germ cells in these *Rspo1* mutants [6]. The increase of apoptotic cells observed in another XX *Rspo1^−/−^* gonads could be due either to a different genetic background or a different mutant allele [43]. Thus depletion in germ cell number in XX *Rspo1^−/−^* mutants is not due to apoptosis, but must be caused by defects in germ cell proliferation.

### Loss of *Rspo1* impairs XX germ cell meiosis

**Figure 2:**
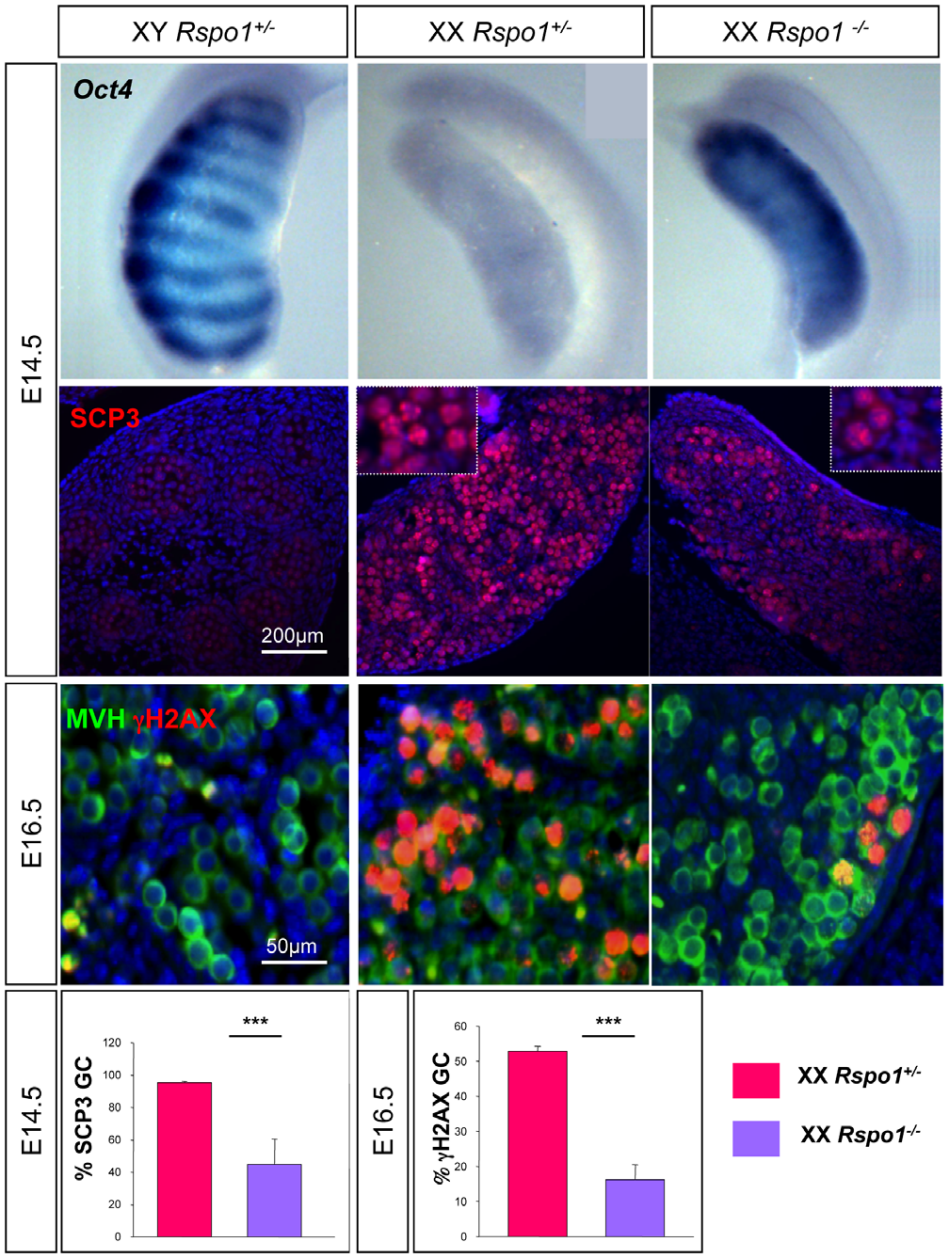
*Rspo1* promotes XX germ cells meiosis. Upper panel: *Oct4* expression is maintained in XX *Rspo1* mutant gonads. *Oct4* whole-mount *in situ* hybridization at E14.5 in XY, XX control and XX *Rspo1^−/−^* fetal gonads. Middle panels: Downregulation of meiotic markers in XX *Rspo1* mutant gonads. Immunodetection of SCP3 in E14.5 gonads and of γH2AX and MVH in E16.5 gonads. DAPI (blue): nuclei. Lower panel: Quantification of meiotic germ cells in XX *Rspo1^+/−^* and *Rspo1^−/−^* gonads at E14.5 (SCP3) and E16.5 (γH2AX). Bars represent mean+1 SEM, n = 15 sections of each genotype.

After proliferation, XX fetal germ cells enter meiosis at around E13.5–E14.5 whereas male germ cells arrest in G0/G1 phase of the cell cycle At E14.5, when female germ cells enter meiosis, the expression of *Oct4* is down-regulated in ovaries whereas it is maintained in XY gonads [44], [45], [46]. *In situ* hybridization studies for *Oct4* at E14.5 revealed robust expression in XX *Rspo1^−/−^* gonads and XY gonads, but not in XX *Rspo1^+/−^* (Fig. 2) suggesting that RSPO1 is required for *Oct4* down-regulation in XX germ cells. We therefore asked whether entry into meiosis was occurring normally in XX *Rspo1^−/−^* gonads. Immunostaining using two meiotic markers, SCP3 and γH2AX, demonstrated a dramatically reduced number of meiotic cells in XX *Rspo1^−/−^* compared to XX *Rspo1^+/−^* gonads at E14.5 and E16.5 (Fig. 2). Quantification of the number of meiotic germ cells (SCP3 or γH2AX positive) demonstrated that germ cells in XX *Rspo1^−/−^* gonads failed to enter meiosis (Fig. 2). The number of meiotic germ cells varied between XX *Rspo1^−/−^* gonads, but in all cases, at least half of the germ cells did not initiate meiosis. The fact that some germ cells are still able to enter meiosis in XX *Rspo1^−/−^* gonads suggests that *Rspo1* is not absolutely required for entry into meiosis. All together, this suggests that *Rspo1* is involved in expression of meiotic genes and entry into meiosis in XX germ cells.

### RSPO1 contributes to *Stra8* expression

**Figure 3:**
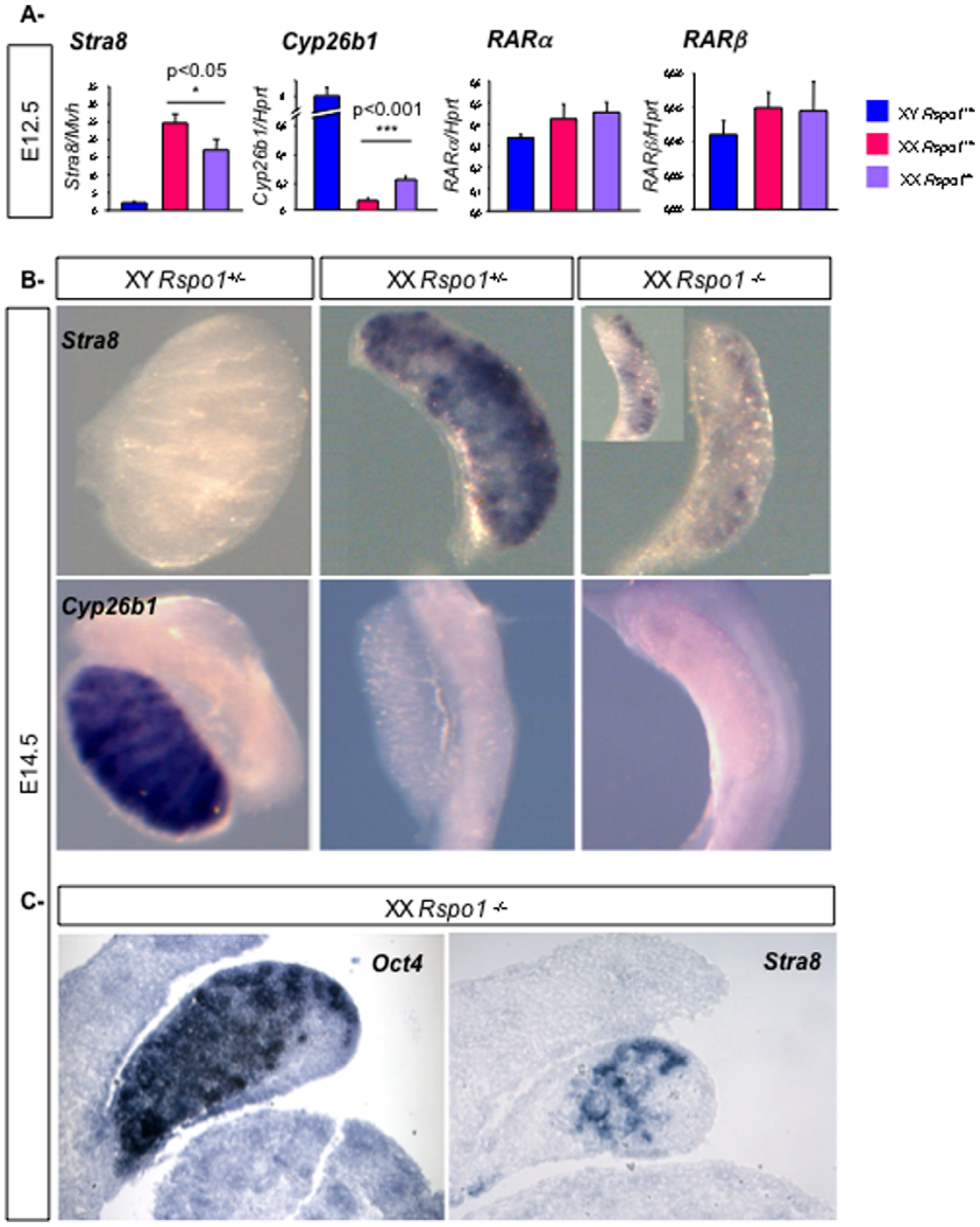
*Rspo1* is involved in induction of *Stra8* expression. A- Quantitative RT-PCR analysis of *Stra8*, *Cyp26b1*, *RARα* and *RARβ* expression in E12.5 XY control, XX control, and *Rspo1^−/−^* gonads, using *Mvh* (for *Stra8* qPCR) or *Hprt* (for other qPCR) as the normalization control. Bars represent mean+1 SEM, n = 8 individual embryos. B- *In situ* hybridizations at E14.5 in XY and XX control and XX *Rspo1^−/−^* gonads for *Stra8* and *Cyp26b1*. C- *In situ* hybridizations on sections of the same XX *Rspo1^−/−^* gonad for *Oct4* and *Stra8* at E14.5.

Entry into meiosis is sensitive to the genetic background [37]. Indeed, a function of *Dazl* in the initiation of meiosis in fetal ovaries could only be unravelled on a pure C57BL6 background. *Stra8* (*Stimulated by Retinoic Acid 8*) is essential for meiosis induction (Baltus et al. 2006) and previously, we observed a small but not significant down-regulation of *Stra8* expression (Fig. 3 in [6]). To know the relevance of this down-regulation, *Stra8* expression was studied in XX *Rspo1^−/−^* gonads with a higher content of C57BL6/J. It has been shown that RA induces *Stra8* expression in germ cells in XX gonads [22], [23]. However, a recent report that *Stra8* expression is induced normally in XX gonads from XX *Raldh2^−/−^* embryos that lack any detectable RA in their urogenital ridges, has led to the suggestion that *Stra8* expression can be induced independently of RA in this developmental context [25]. This suggests that other signaling pathways might also be able to induce *Stra8* expression in fetal female germ cells. To address whether *Rspo1* could be involved in *Stra8* expression, we analysed *Stra8* expression levels in XX *Rspo1^−/−^* gonads in comparison to controls. In the ovary, *Stra8* expression begins from E12.5. Quantitative real-time RTPCR (Q-PCR) and *in situ* hybridization experiments showed that *Stra8* expression was reduced at E12.5 and E14.5 respectively (Fig. 3A–B) when compared to XX *Rspo1^+/−^* gonads. Although *Stra8* expression was consistently down regulated, the extent of this down regulation varied between embryos (see Inset Fig. 3B). In addition, *in situ* hybridization experiments on sections showed that *Oct4* and *Stra8* positive cells were present in the same XX *Rspo1^−/−^* gonads (Fig. 3C) suggesting that germ cells did not enter meiosis and stayed undifferentiated. However, the relatively small decrease in *Stra8* expression in XX *Rspo1^−/−^* gonads at E12.5 suggests that RSPO1 is not the main/only regulator of *Stra8* expression. Nevertheless, *Rspo1* does play a role in inducing *Stra8* expression in female fetal germ cells.

In the embryonic testis, meiosis inhibition and *Stra8* repression are at least partially regulated via CYP26B1, an enzyme that metabolizes RA [25], [36]. Measurements of *Cyp26b1* expression levels at E12.5 showed these remained higher in XX *Rspo1^−/−^* gonads when compared to XX controls. Although at E12.5, *Cyp26b1* expression was 40 fold lower than in XY gonads, we cannot exclude that CYP26B1 is involved in *Stra8* down-regulation in XX *Rspo1* mutants. However, *in situ* hybridization analysis showed that *Cyp26b1* was weakly or not expressed in XX *Rspo1^−/−^* gonads at E14.5 or at E16.5 (Fig. 3B & data not shown), when compared to XY gonads. At these stages, *Stra8* and other meiotic markers (Fig. 3A and Fig. 2) were strongly down-regulated suggesting that *Cyp26b1* is not the main factor repressing *Stra8* expression in XX *Rspo1^−/−^* gonads at E14.5.

*Stra8* expression can be induced by RA treatments [47]. RA acts by binding to the Retinoic Acid Receptors (RAR), which in turns bind to retinoic acid response elements (RAREs) in the regulatory regions of direct target genes [48]. One of these direct targets is *RARβ*, and thus *RARβ* expression is up-regulated by RA signaling [49]. We next addressed whether the RA signaling pathway is affected in XX *Rspo1^−/−^* gonads by quantification of the level of expression of *RARa* and *β* using Q-PCR experiments. At E12.5, *RARa* and *RARβ* levels were not changed between the different genotypes (Fig. 3A), but the variability in *RAR* expression levels do not allow us to completely exclude small differences in *RAR* expression levels in XX *Rspo1^−/−^* gonads”. However, these results suggest that *Stra8* induction by *Rspo1* is not dependent on RA signaling.

### Sex reversal of germ cells in XX *Rspo1^−/−^* gonads

**Figure 4:**
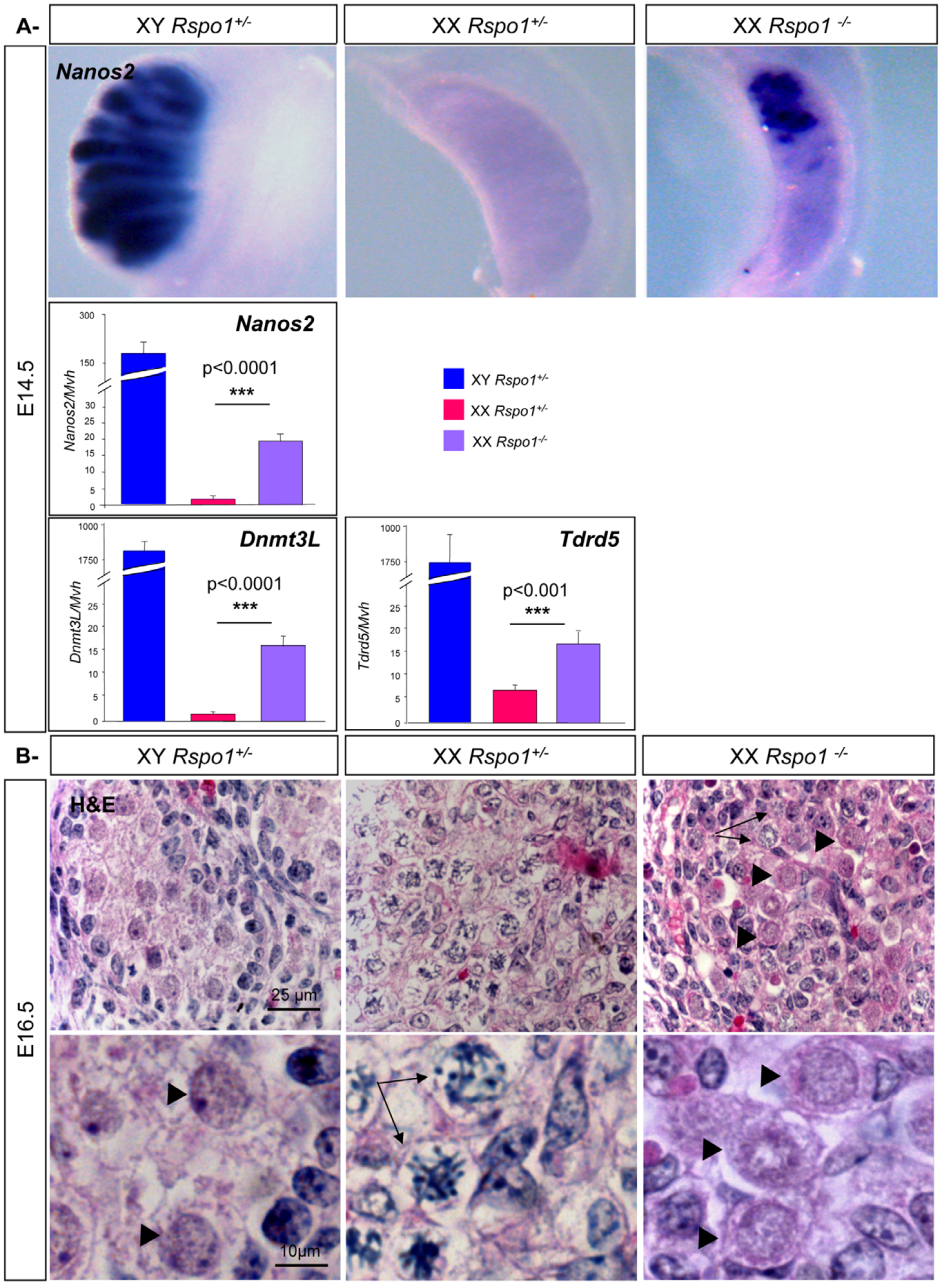
Up-regulation of of gonocyte markers in XX *Rspo1* mutant gonads. A- *In situ* hybridizations at E14.5 in XY and XX control and XX *Rspo1^−/−^* gonads for *Nanos2*. Histograms: Quantitative RT-PCR analysis of *Nanos2*, *Dnmt3L* and *Tdrd5* expression in E14.5 XY control, XX control and *Rspo1^−/−^* gonads, using *Hprt* as the normalization control. Bars represent mean+1 SEM, n = 8 individual embryos. B- Haematoxylin & Eosin staining of gonadic sections of XY, XX *Rspo1^+/−^* and XX *Rspo1^−/−^* gonads at E16.5. Meiotic oogonia are indicated by arrows GO/G1 quiescent gonocytes are indicated by arrowheads.

To investigate how germ cells differentiate in XX *Rspo1^−/−^* mutants, we analysed the expression of known gonocyte makers. In wild type mice, *Nanos2* is expressed specifically in male gonocytes. Moreover, transgenic analysis showed that *Nanos2* is sufficient to repress *Stra8* expression, prevent meiosis, and induce XX germ cells to adopt a male fate [39]. *In situ* hybridization and Q-PCR analyses demonstrated strong expression of *Nanos2* in XX germ cells of *Rspo1^−/−^* gonads compared to XX controls at E14.5 whereas *Nanos2* expression was not detectable at E12.5 ((Fig. 4A) and data not shown). The localization of *Nanos2* expression was variable and not always at the anterior pole in the XX *Rspo1^−/−^* animals (data not shown), a pattern likely associated with the variability of the genetic background. The upregulation of *Nanos2* in XX *Rspo1^−/−^* gonads was around 10-fold lower than XY control gonads which is consistent with *in situ* hybridization data showing that only some germ cells in XX *Rspo1^−/−^* gonads express high levels of *Nanos2*. This ectopic activation of *Nanos2* provides a possible explanation for the partial repression of *Stra8* expression and meiosis inhibition observed in XX *Rspo1^−/−^* gonads at E14.5. Moreover, we quantified the level of expression of other gonocyte markers such as *Dnmt3L* and *Tdrd5*. Q-PCR analyses showed that their expression was significantly up-regulated in the XX *Rspo1^−/−^* gonads compared to XX control gonads at E14.5 (Fig. 4A). However, their level of expression was 100 fold lower in XX *Rspo1* mutant gonads versus XY gonads suggesting that only some of the germ cells in the mutant gonads followed a male differentiation pathway or that progression along the male differentiation pathway is impaired or delayed, in comparison to gonocytes in XY gonads. These cells, representing about 50% of the germ cells in the mutant gonads, may stay in an indifferentiated/pluripotent state as suggested by the maintenance of *Oct4* expression observed in *in situ* hybridization experiments at E14.5 (Fig. 2).

These results were illustrated by histological analyses of gonads at E16.5 (Fig. 4B), when all the female germ cells have normally entered meiosis. Indeed, germ cell chimaerism was observed in XX *Rspo1^−/−^* gonads with germ cells that have initiated meiosis as expected from oocytes (Fig. 4B - arrows) and others which were comparable to quiescent gonocytes in XY gonads (Fig. 4B - arrowheads) further supporting that a certain proportion of germ cells were sex reversed in XX *Rspo1^−/−^* gonads. At this stage, these XX gonocytes were single, isolated cells and not enclosed by sex cords in XX mutant gonads.

### Germ cell proliferation and meiosis defects are independent of Sertoli cells in XX *Rspo1^−/−^* gonads

**Figure 5:**
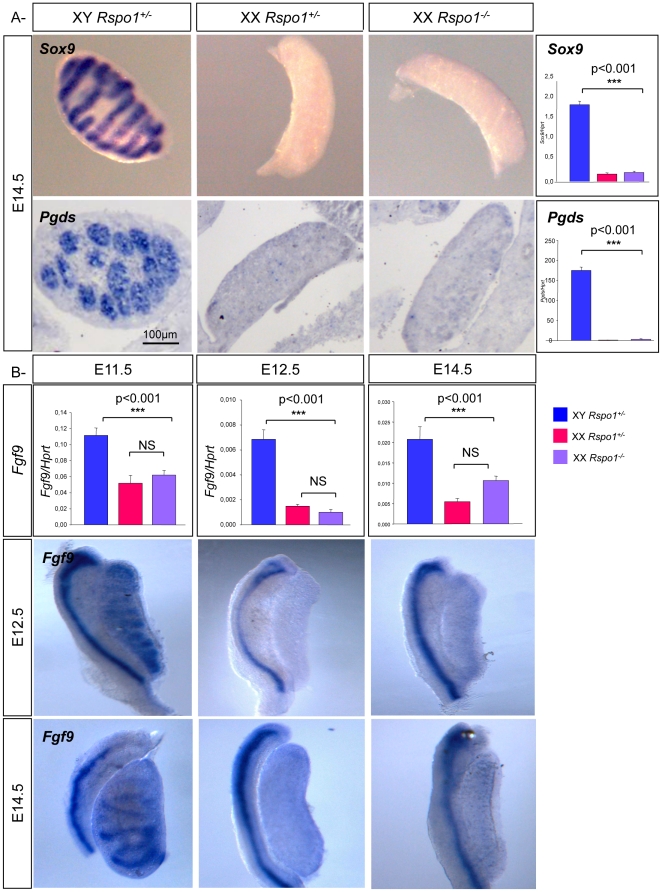
Germ cell sex reversal is not caused by Sertoli cell factors in XX *Rspo1* mutant gonads. A- Lack of Sertoli cell differentiation at E14.5 in XX *Rspo1* mutant gonads. *In situ* hybridizations of *Sox9* and *Pgds* in E14.5 XY and XX control and XX *Rspo1^−/−^* gonads. Right panels: Quantitative RT-PCR analysis of *Sox9* and *Pgds*, using *Hprt* as the normalization control. Bars represent mean+1 SEM, n = 8 individual embryos. B- *Fgf9* is not significantly expressed at E11.5 and E12.5 in XX *Rspo1* mutant gonads. Upper panels: Quantitative RT-PCR analysis of *Fgf9* at E11.5, E12.5 and E14.5 using *Hprt* as the normalization control. NS: Not significant. Bars represent mean+1 SEM, n = 8 individual embryos. Lower panels: *In situ* hybridizations of *Fgf9* (E12.5 and E14.5) in XY and XX control and XX *Rspo1^−/−^* gonads.

The germ cell proliferation defect and meiotic blockage that we observed in XX *Rspo1^−/−^* gonads could be either a consequence of supporting cell sex reversal in these gonads, or could reflect a more direct role for *Rspo1* in promoting proliferation and meiosis in fetal germ cells. In order to check whether the supporting cells sex reversed and differentiated into Sertoli cells before sex reversal of the germ cells in XX *Rspo1^−/−^* gonads, we performed Q-PCR analysis for the Sertoli cell markers *Sox9* and *Pgds*. These markers were slightly increased in XX *Rspo1^−/−^* gonads compared to the XX *Rspo1^+/−^* gonads at E12.5 (data not shown) and E14.5, but this increase was much lower than the high expression levels of these genes observed in XY gonads (Fig. 5A, right panels). To check whether Sertoli cells were present in XX *Rspo1^−/−^* gonads at E14.5, we performed *in situ* analysis using the Sertoli cell markers *Sox9*, *Pdgs*, SDMG1 and *Sox8* (Fig. 5A & data not shown). While these genes were highly expressed in XY gonads, no significant expression could be detected in XX *Rspo1^−/−^* mutants by *in situ* hybridization. *Sox9* expression and Sertoli cell differentiation did eventually occur in XX *Rspo1^−/−^* gonads by E18.5 ([6] & data not shown), but the prevention of meiosis and changes in germ cell gene expression seen by E14.5 in XX *Rspo1^−/−^* gonads preceded detectable Sertoli cell differentiation.

### Meiosis defects are independent of *Fgf9* expression in XX *Rspo1^−/−^* gonads

Deletion of *Fgf9* induces massive germ cell loss in fetal XY gonads and about half of the remaining germ cells enter meiosis [50]. Furthermore, FGF9 inhibits *Stra8* up-regulation and entry into meiosis [35], [36]. Although *Fgf9* is up-regulated in Sertoli cells of XY gonads, it is initially expressed in both, XX and XY undifferentiated gonads [33]. We therefore asked whether ablation of *Rspo1* would allow gonads to express high levels of *Fgf9* that in turn could inhibit meiosis. Previous Q-PCR analysis showed an increase of *Fgf9* expression at E14.5 [6]. However, new Q-PCR analyses at E11.5, E12.5 and E14.5 did not show significant increase in *Fgf9* levels in XX control versus XX *Rspo1^−/−^* gonads. *Fgf9* is expressed in mesonephric tubules which are closely linked to the developing gonads and we cannot exclude that some *Fgf9* signals come from these mesonephric cells. To check these results, *in situ* hybridization experiments were carried out and showed no differences in staining between XX control and XX *Rspo1^−/−^* gonads at E12.5 and E14.5 (Fig. 5B).

It has been shown previously that *Rspo1* is genetically up-stream of *Wnt4* during ovarian development and *Rspo1* is required for *Wnt4* up-regulation in the XX gonad. Since *Wnt4* expression is antagonistic of *Fgf9* expression [33], [51], it could be expected that RSPO1 would indirectly prevent *Fgf9* expression. However, *Rspo1* depletion did not trigger robust *Fgf9* expression suggesting that the remaining expression of *Wnt4* in XX *Rspo1^−/−^* gonads, is sufficient to prevent *Fgf9* expression. In addition, it is not known whether other pathways, independent of the *Wnt4* pathway and potentially involved in *Fgf9* regulation, are activated by RSPO1. In summary, the failure to enter meiosis does not seem to be caused by an increase of *Fgf9* expression in XX *Rspo1^−/−^* gonads but rather suggests a direct effect of RSPO1 promoting germ cells to differentiate along the female pathway.

### RSPO1 regulates the transcriptional activity of β-catenin in XX germ cells

*R-spondins* are activators of the canonical WNT/β-catenin signaling and *Rspo1* activates this pathway to promote ovarian differentiation [6], [11], [12], [13]. Recent data show that the secreted RSPO1 protein localizes at the plasma membrane of not only somatic, but also germ cells [41], [42] suggesting that RSPO1 may be able to activate β-catenin directly in germ cells.

**Figure 6:**
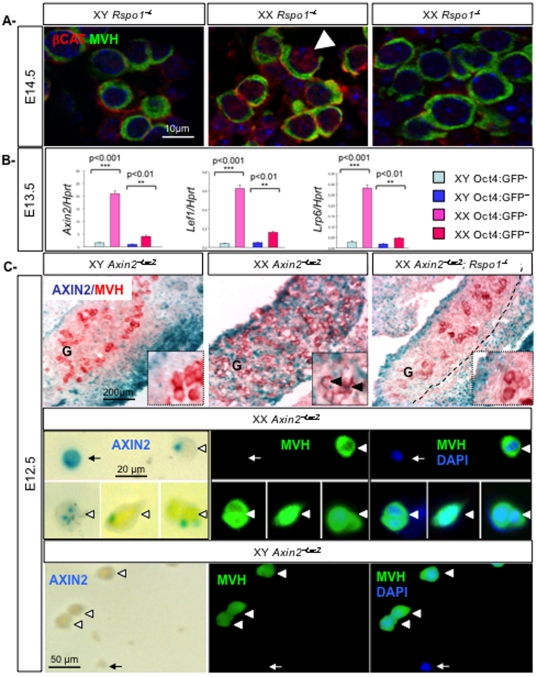
*Rspo1* activates β-catenin signaling pathway in XX germ cells. A- Stabilization of β-catenin in XX germ cells is mediated by *Rspo1*. Active β-catenin (red) and MVH immunostaining (germ cells, green) in XY, XX *Rspo1^+/−^* and *Rspo1^−/−^* gonads at E14.5. DAPI (blue): nuclei. Arrowheads: nuclear β-catenin staining. B- Effectors of the WNT/β-catenin signaling pathway are expressed in XX germ cells. Quantitative RT-PCR analysis of *Axin2*, *Lef1*, *LRP6* expression in E13.5 germ cells (XX and XY *Oct4*-positive cells) and somatic cells (XX and XY *Oct4*-negative cells), using respectively *Hprt* as the normalization control. Bars represent mean+1 SEM, n = 3 individual experiments. C- Expression of *Axin2*, a target of β-catenin in XX germ cells. X-Gal staining (AXIN2) (blue) and MVH immunostaining (germ cells, red) in XY *Axin2^+/LacZ^*, XX *Axin2^+/LacZ^* and XX *Axin2^+/LacZ^*; *Rspo1^−/−^* gonads at E12.5. Arrowheads: germ cells. G: gonad. X-Gal staining (AXIN2) (blue) and MVH immunostaining (germ cells, green) on isolated germ cells from XY *Axin2^+/LacZ^* and XX *Axin2^+/LacZ^* gonads at E12.5. Arrowheads: germ cells, arrows: somatic cells.

To investigate whether the WNT/β-catenin signaling pathway is active in fetal germ cells, we performed immunostaining at E14.5. Stabilized β-catenin was mainly found in the nuclei of XX somatic and germ cells whereas in XY gonads it predominantly localized to the cellular membranes (Fig. 6A)(see also Fig.6 in [6]). Since nuclear staining was weaker or absent in both XY and in XX *Rspo1^−/−^* germ cell nuclei (Fig. 6A), we conclude that RSPO1 is required for the sex specific activation of β-catenin in XX germ cells at this stage.

The nuclear form of β-catenin has a transcriptional function and together with TCF/LEF transcription factors induces expression of target genes like *Axin2* and *Lef1* [52], [53]. To confirm a sex specific activation of **β**-catenin signalling, we next investigated whether effectors of this signaling pathway were expressed in fetal germ cells. For this, we purified germ cells using FACS sorting from E13.5 *Oct4-GFP* transgenic gonads [54], a transgene that is specifically expressed in germ cells but not in somatic cells of the gonad. Q-PCR experiments were used to quantify the expression levels of known WNT/β-catenin partners and showed that *Axin2*, *Lef1* and *LRP6* (the RSPO1 receptor) [11] were strongly expressed in XX somatic cells. This is in agreement with our previous study showing that RSPO1 is involved in β-catenin activation in ovarian somatic cells [6]. In addition, *Axin2*, *Lef1* and *LRP6* were also expressed in XX germ cells but at a lower level then in somatic tissues (Fig. 6B). Importantly, levels of the markers of β-catenin signaling, *Axin2*, *Lef1* and *LRP6*, were significantly higher in XX germ cells compared to XY germ cells. Analyses of the expression levels of *Mvh* (germ cell marker), *Sf1* (highly expressed in male somatic cells at E13.5), *Bmp2*, *Fst* and *Rspo1* (female somatic cell markers) confirmed that there was no significant contamination of germ cells by somatic cells (Fig.S1). Activation of β-catenin was expected in XY somatic cells from the urogenital ridges as evidenced by expression of *Axin2* (Fig.S1) since the WNT/β-catenin signalling pathway has been shown to be active in the Mullerian duct [55]. Taken together, these data show that members of the canonical β-catenin signaling pathway are expressed in XX germ cells, suggesting that this pathway is also functional within germ cells.

We had previously investigated the transcriptional function of β-catenin in fetal gonads at E14.5 using the *Axin2^+/LacZ^* line that is considered to be a universal reporter of the canonical β-catenin signaling pathway [6], [52]. Strong β-galactosidase staining had been previously observed in the somatic cells in XX fetal gonads but such a staining was not easily detectable in germ cells [6]. However, detailed examination of gonads from XX and XY *Rspo1^+/−^*; *Axin2^+/LacZ^* or *Rspo1^−/−^*; *Axin2^+/LacZ^* embryos at E12.5 revealed that, in addition to strong β-galactosidase staining in somatic cells, weaker β-galactosidase staining was present as a cytoplasmic dot in XX germ cells (Fig. 6C). This cytoplasmic dot of β-galactosidase staining was not present in germ cells from XY gonads or from XX *Rspo1^−/−^* gonads. To confirm that the cytoplasmic dot of β-galactosidase staining was localized within germ cells, we purified germ cells from XX and XY *Axin2^+/LacZ^* gonads at E12.5, when germ cells are highly proliferative. Subsequently, we found cytoplasmic dots of β-galactosidase staining both in XX somatic and germ cells, but not in XY germ cells (Fig. 6C). Cytoplasmic dots of β-galactosidase staining in XX germ cells have previously been observed in other transgenic lines that express the *LacZ* reporter in germ cells [56], [57]. The biological basis of the localization of this staining remains to be explored and could be mediated by cryptic or cell-type specific post-transcriptional processing of the *LacZ* reporter that targets the β-galactosidase protein to a cytoplasmic structure such as the Golgi apparatus. Nevertheless, the presence of RSPO1-dependent β-galactosidase staining in XX germ cells suggests that the *Axin2-LacZ* reporter has been transcriptionally activated in these cells and indicates that active β-catenin signaling is present in XX germ cells at E12.5.

Taken together these data suggest that RSPO1 is responsible for the activation of the WNT/β-catenin signaling pathway in XX germ cells during embryogenesis. Since RSPO1 is produced and secreted by somatic cells, it activates the β-catenin signaling pathway in female germ cells in a paracrine manner.

### Ectopic expression of β-catenin prevents quiescence of XY germ cells

**Figure 7:**
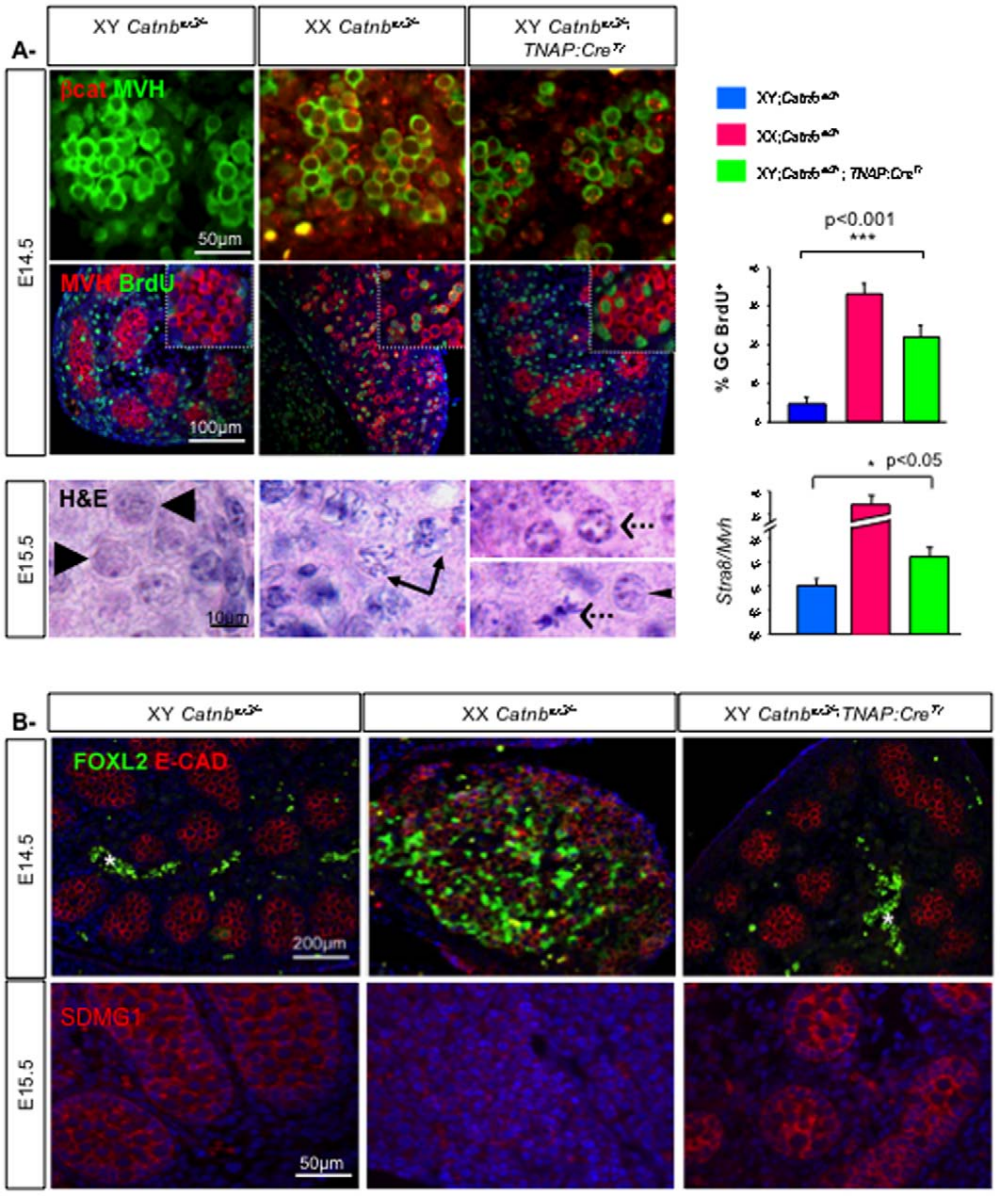
Activation of β-catenin promotes XY germ cell proliferation in fetal gonads. A- Upper panel: Immunodetection of active β-catenin and MVH in XY and XX control and XY *Catnb^ex3/+^*; *TNAP:Cre^Tr^* gonads at E14.5. Middle panel: Immunodetection of the proliferating germ cells with BrdU and MVH in XY and XX control, and XY *Catnb^ex3/+^*; *TNAP:Cre^Tr^* gonads at E14.5. DAPI (blue): nuclei. Histograms: Percentage of BrdU-positive germ cells in XY and XX control, and XY *Catnb^ex3/+^*; *TNAP:Cre^Tr^* gonads at E14.5. Bars represent mean+1 SEM, n = 24 sections of each genotype. Lower panel: Haematoxylin & Eosin staining of E15.5 gonadic sections from XY and XX wild type gonads and XY *Catnb^ex3/+^*; *TNAP:Cre^Tr^* gonads. Arrowheads: XY quiescent gonocytes, arrows: meiotic germ cells, dashed arrows: mitotic germ cells, thin arrowhead: putative early meiotic germ cell. Histogram: Quantitative RT-PCR analysis of *Stra8* at E15.5 using *Mvh* as the normalization control. Bars represent mean+1 SEM, n = 8 individual embryos. B- Upper panel: Immunostaining of FOXL2 (green) and E-Cadherin (germ cells, red) in XY and XX control, and XY *Catnb^ex3/+^*; *TNAP:Cre^Tr^* gonads at E14.5. The white star indicates fluorescent blood cells within the coelomic vessel of the XY gonads. Lower panel: Immunodetection of the Sertoli cell marker SDMG1 in the same gonads. DAPI (blue): nuclei.

In male fetal mice, gonocytes proliferate until about E14.5, when they exit the cell cycle and become quiescent [19]. If β-catenin is required for female germ cell proliferation, we can expect that an ectopic activation of this gene in XY embryonic germ cells prevents gonocytes from becoming quiescent. To test this hypothesis, we generated embryos carrying a conditional constitutively active allele of β-catenin (*Catnb^ex3^*) [58] in combination with the *TNAP:Cre* transgene that drives expression of the CRE recombinase in primordial germ cells from E9.5–10.5 until E15.5 and some somatic tissues [59]. Ectopic activation of β-catenin in XY germ cells was confirmed by immunostaining. At E14.5, we detected nuclear β-catenin in XY germ cells and some surrounding somatic cells in *Catnb^ex3/+^*; *TNAP:Cre^Tr^* gonads whereas XY wild type germ cells were devoid of such a staining (Fig. 7A). To measure the proliferation of germ cells, we carried out BrdU incorporation experiments at E14.5. The percentage of germ cells that incorporated BrdU was dramatically increased in XY *Catnb^ex3/+^*; *TNAP:Cre^Tr^* compared to XY wild type gonads (Fig. 7A). Moreover, at E15.5 and E17.5, germ cells in *Catnb^ex3/+^*; *TNAP:Cre^Tr^* embryonic testes were not quiescent ((Fig. 7A) and data not shown respectively), as evidenced by the continuing presence of condensed chromosomal figures: 16% to 43% (6 gonads) of germ cells in *Catnb^ex3/+^*; *TNAP:Cre^Tr^* mice were in mitotic prophase through telophase, compared to 4% in XY wild types (2 gonads) at E15.5. A small proportion of germ cells showed signs of chromosome condensation indicative of eventual early meiosis (Fig. 7A). Q-PCR experiments showed that *Stra8* expression was up-regulated in XY *Catnb^ex3/+^*; *TNAP:Cre^Tr^* gonads (Fig. 7A). However immunofluorescent analysis with anti-SCP3 antibody, an early meiotic marker, suggested that meiosis was either not occurring or not progressing normally in these cells, and no decrease of *Nanos 2* expression in the *Catnb^ex3/+^*; *TNAP:Cre^Tr^* embryonic testes could be detected at E15.5 (data not shown).

The *TNAP:Cre* transgene expression is not restricted to germ cells and can lead to ectopic activation of β-catenin in XY somatic cells. It has been previously shown that ectopic stabilization of β-catenin in the somatic cells can induce sex reversal of XY gonads [9]. However, the ovarian marker FOXL2 was not expressed in XY *Catnb^ex3/+^*; *TNAP:Cre^Tr^* somatic cells in contrast to XX control gonads (Fig. 7B) indicating that XY *Catnb^ex3/+^*; *TNAP:Cre^Tr^* gonads were not sex-reversed. Testis differentiation in XY *Catnb^ex3/+^*; *TNAP:Cre^Tr^* gonads was confirmed by immunostaining with SDMG1, a marker of Sertoli cells at E15.5 (Fig. 7B). This suggests that the expression of β-catenin in somatic cells of XY gonads, promoted by the *TNAP:Cre* transgene, is not sufficient to change the somatic fate. Altogether, this indicates that β-catenin activation in XY germ cells is sufficient to induce cell cycle progression.

### Somatic ablation of β-catenin does not affect germ cell proliferation and differentiation

To determine which aspects of the XX *Rspo1^−/−^* phenotype are caused by loss of active β-catenin signaling in gonadal somatic cells, and which aspects of the XX *Rspo1^−/−^* phenotype are caused by loss of active β-catenin signaling in the germ cells, we used tissue-specific *Cre* lines to specifically delete β-catenin in either the germ cells or gonadal somatic cells. Specific ablation of β-catenin in germ cells using the *TNAP:Cre* transgenic line caused embryonic death, a phenotype that is likely due to leaky expression of the *Cre* recombinase in some somatic tissues at early stages of development [60] when β-catenin is essential [61]. To know whether the germ cell defects seen in XX *Rspo1^−/−^* gonads could be a consequence of β-catenin signaling in somatic cells, we carried out genetic crosses to conditionally knock out β-catenin signaling pathway in somatic cells. This was achieved using the *Sf1:Cre* line [62], which is expressed from E10.5–11.5 in the gonadal primordium. Depletion of β-catenin in XX *Catnb^flox/flox^*; *Sf1:Cre^Tr^* gonads impairs ovarian somatic cell differentiation as evidenced by down-regulation of *Wnt4* and *Fst*, two genes expressed in the ovarian supporting cells [63].

**Figure 8:**
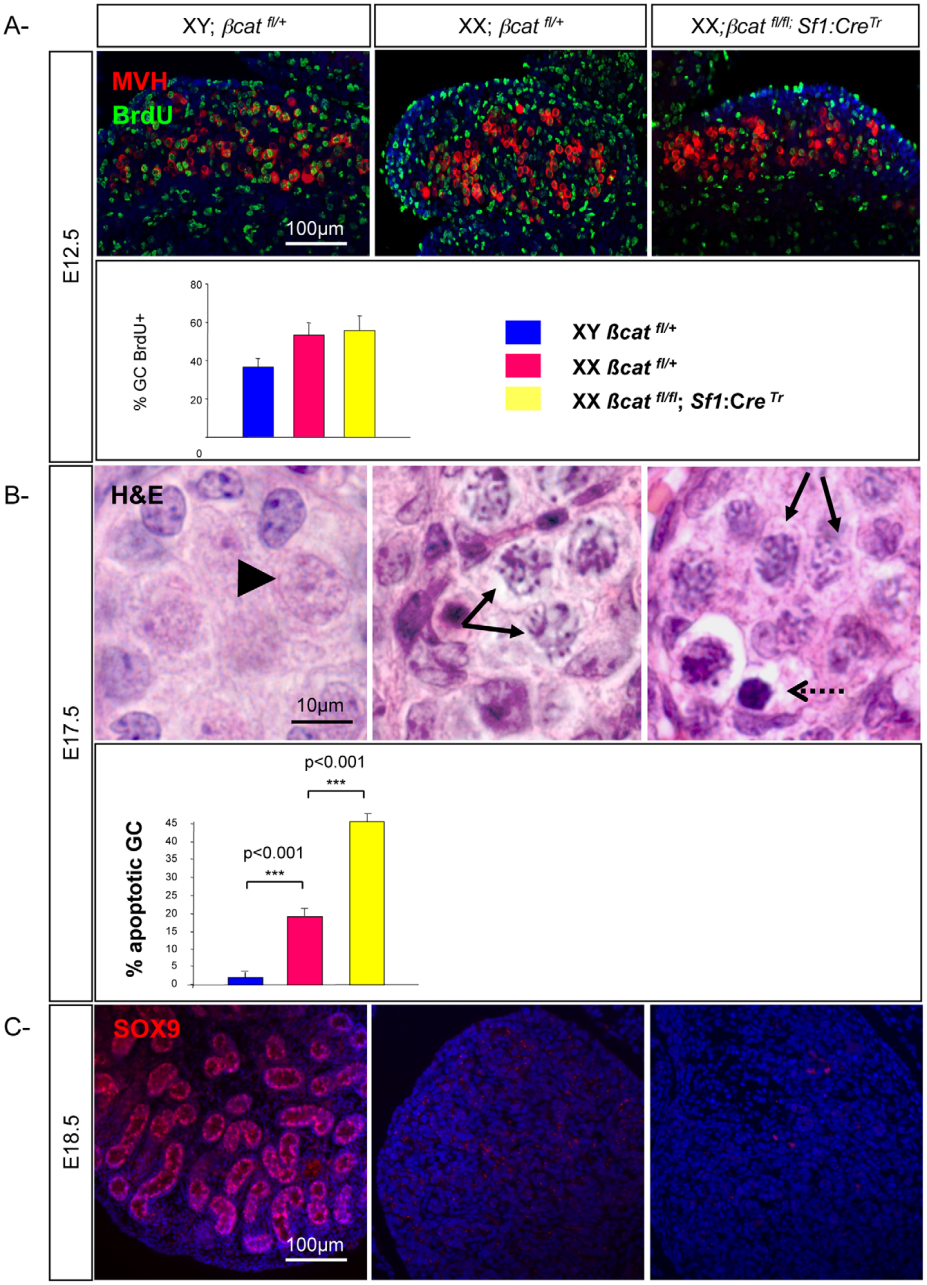
Somatic ablation of β-catenin does not affect germ cell fate. A- Proliferation is not impaired in XX *Catnb ^flox/flox^*; *Sf1:Cre^Tr^* gonads. Immunodetection of the proliferating germ cells with BrdU (green) and MVH (red) in XY, XX control and XX *Catnb ^flox/flox^*; *Sf1:Cre^Tr^* gonads at E12.5. DAPI (blue): nuclei. Histograms: Percentage of BrdU-positive germ cells in XY, XX control and XX *Catnb ^flox/flox^*; *Sf1:Cre^Tr^* gonads at E12.5. Bars represent mean+1 SEM, n = 24 sections of each genotype. B- Meiosis in not hampered in XX*Catnb ^flox/flox^*; *Sf1:Cre^Tr^* gonads. Haematoxylin & Eosin staining of sections from E17.5 XY, XX control and XX *Catnb ^flox/flox^*; *Sf1:Cre^Tr^* gonads. Arrowheads: gonocytes, arrows: meiotic germ cells, dotted arrows: apoptotic germ cells. *Histograms*: Percentage of apoptotic germ cells versus total germ cells after TUNEL staining in XY, XX control gonads and XX *Catnb ^flox/flox^*; *Sf1:Cre^Tr^* gonads. Bars represent mean+1 SEM, n = 24 sections of each genotype. C: Immunodetection of SOX9 (red) in XY, XX control gonads and XX *Catnb ^flox/flox^*; *Sf1:Cre^Tr^* gonads at E18.5. DAPI (blue) was used to detect nuclei.

We performed BrdU incorporation experiments, and quantified the number of proliferating germ cells in XY, XX controls and XX *Catnb^flox/flox^*; *Sf1:Cre^Tr^* gonads. Statistical analyses showed that the percentage of proliferating germ cells was similar in XX *Catnb^flox/flox^*; *Sf1:Cre^Tr^* gonads when compared to XX control gonads (Fig. 8A), suggesting that depletion of β-catenin activation in *Sf1*-positive cells does not impair germ cell proliferation. Moreover, histological analysis demonstrated that while XY gonocytes were arrested in G0/G1 phase, oogonia in both wild type XX and XX *Catnb^flox/flox^*; *Sf1:Cre^Tr^* gonads initiated meiosis before becoming apoptotic (Fig. 8B). These data and others [63] showed that depletion of β-catenin activation in *Sf1*-positive somatic cells impairs ovarian somatic differentiation but does not affect germ cell proliferation and meiosis.

Altogether, these data suggest that defects in germ cell proliferation and meiosis in XX *Rspo1^−/−^* gonads are not an indirect consequence of ablating β-catenin signaling in the somatic cells, but are rather due to a direct action of RSPO1 to activate β-catenin signaling within germ cells to promote oogenesis in the fetal ovary.

## Discussion

In mice, germ cell differentiation is influenced by differentiation of the somatic environment. Although XX and XY germ cells go through similar processes of maturation (proliferation and meiosis), they are regulated in a sex-specific manner. *Rspo1* encodes an activator of the WNT/β-catenin signaling pathway that is secreted by gonadal somatic cells and is required for ovarian differentiation [6], [40], [43]. In ovaries, RSPO1 is up-regulated from E11.5 onwards [7]. In previous studies, we have shown that loss-of-function of *Rspo1* induces not only somatic sex reversal but also germ cell masculinization [6]. In XX *Rspo1^−/−^* gonads, cell-cell adhesions involving germ cells show a similar organization as in XY gonads. These adherens junctions are lost when XX germ cell enter meiosis in ovaries [64]. This seems to be linked to the down-regulation of *Wnt4* expression since this phenomenon has also been described in XX *Wnt4^−/−^* gonads [65]. In addition, germ cell sex reversal was observed in XX *Rspo1^−/−^* gonads [6]. We now have shown that germ cell defects in XX *Rspo1* mutant gonads are independent of Sertoli cell differentiation. The first testis-like structures that appear in XX *Rspo1^−/−^* gonads are the coelomic vessels and steroidogenic lineages expressing *Cyp11a1-P450Scc* [6]. Although there is no evidence suggesting that endothelial or steroidogenic cells are involved in germ cell proliferation and meiotic defects in XX *Rspo1^−/−^* gonads, we cannot exclude completely such a hypothesis.

In the embryonic ovary, germ cells are firstly dividing before entering meiosis. Primordial germ cell proliferate during their migration towards the gonads in both sexes [66]. Once within the gonads, the germ cell intrinsic factor RNA helicase MVH is only required for XY germ cell proliferation while it is expressed in germ cells of both sexes [18]. Indeed, XX germ cells in a testicular environment do not proliferate as efficiently as XY germ cells in the testis or XX germ cells in the ovary [67]. This suggests that sex specific signaling pathways are not only required for sexual differentiation of germ cells [68] but are also involved in germ cell proliferation once they have reached the gonads.

RSPO1 is produced by ovarian somatic cells and induces functional β-catenin activation in germ cells in a paracrine manner. In XX *Rspo1^−/−^* gonads, down-regulation of β-catenin in germ cells is associated with defects of their proliferation, inhibition of meiosis and partial sex-reversal of germ cells. Since *Rspo1* is not expressed in the environment of XY germ cells [7] and β-catenin is not activated in male fetal germ cells, this molecular pathway appears to be female specific. Thus male and female fetal germ cells seem to have acquired independent mechanisms to regulate their proliferation and differentiation, with *Rspo1* and WNT/β-catenin signaling holding a key position within the female lineage.

However, when germ cell proliferation resumes in the postnatal testis prior to entry into meiosis, it is associated with activation of WNT/β-catenin signaling but not with *Rspo1* expression in spermatogonia ([69] and our results (Fig.S2). Indeed, *Axin2^+/LacZ^* reporter expression can be detected in the postnatal XY testis at P0 when germ cells resume mitosis and a robust *Axin2^+/LacZ^* reporter expression is present in XY postnatal testes at 12 days after birth (Fig.S1). Immunostaining with markers for germ cells or Sertoli cells whose cytoplasm surrounds the germ cells at this stage (MVH and SDMG1 respectively) identify the *Axin2^+/LacZ^* positive cells exclusively as germ cells. As parsimony predicts that the same function of β-catenin is conserved in germ cell differentiation in both sexes, WNT/β-catenin signaling may be involved in cell cycle progression in pre-meiotic proliferation in both sexes. Whereas the function of β-catenin remains to be elucidated in spermatogonia, the extracellular signals regulating germ cell proliferation in these tissues are likely to be different.

In addition to proliferation, *Rspo1* is also involved in oogonial differentiation and entry into meiosis. Ectopic activation of β-catenin in XY fetal germ cell increases germ cell proliferation, and prevents germ cells from becoming quiescent, but does not allow them to enter meiosis. Since these mutant germ cells are surrounded by Sertoli cells in a developing testis, Sertoli cell–derived factors such as FGF9 may prevent them from entering meiosis. These conflicting messages acting on the germ cells with β-catenin as a meiosis stimulating factor and Sertoli cell-derived meiosis preventing factors may lead the germ cells to become apoptotic.

In XX gonads, entry into meiosis has been linked to RA signaling, which induces the expression of *Stra8* [22], [23]. However, it has been reported that *Stra8* remains expressed in XX gonads devoid of RA synthesis and signalling and that *Stra8* expression and entry into meiosis may be stimulated by an alternative mesonephros-derived substrate of CYP26b1 [25]. At E12.5, the remaining expression of *Cyp26b1* in XX *Rspo1^−/−^* gonads may participate in *Stra8* repression by metabolizing this mesonephric substrate. Our data suggest that the RSPO1/β-catenin signaling pathway is also involved in entry into meiosis. It would be interesting to investigate whether expression of the RSPO1/β-catenin signaling pathway in the female gonad is induced by the putative *Raldh*-independent meiosis inducer that has been proposed to originate in the mesonephros [25].

The penetrance of the meiotic blockage in XX *Rspo1^−/−^* gonads is not complete indicating that β-catenin is probably not the only germ cell intrinsic factor involved in the regulation of *Stra8* expression, as germ cells in XX *Rspo1^−/−^* gonads enter meiosis. At E14.5, *Nanos2* is expressed in XX *Rspo1^−/−^* gonads suggesting that this meiosis preventing factor [39] is involved in *Stra8* down-regulation at this stage. However, *Nanos2* is not expressed at E12.5 in XX *Rspo1* mutant gonads (data not shown), thus it cannot be involved in the initial repression of *Stra8* and subsequent meiosis blockage nor in proliferation defects.

**Figure 9:**
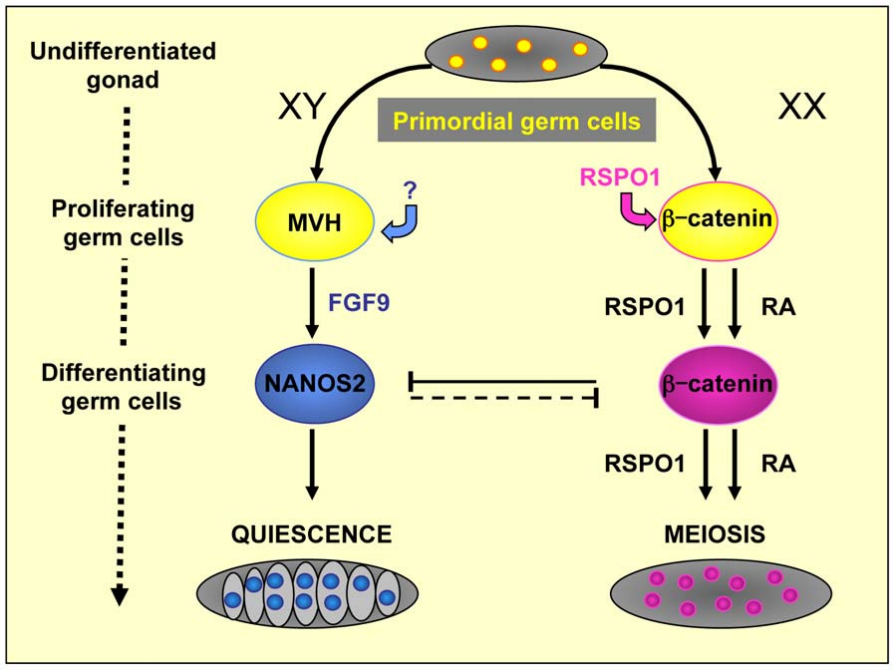
Opposing signals regulate germ cell sexual differentiation. Male and female fetal germ cells have acquired independent mechanisms to regulate their differentiation. In the fetal testis, *Mvh* is required to promote germ cell proliferation. FGF9 secreted by Sertoli cells promotes germ cell survival and may inhibit entry into meiosis. Then *Nanos2* promotes differentiation into gonocytes and meiosis inhibition. RSPO1 has been shown to be a secreted activator of β-catenin. In fetal ovary, RSPO1/β-catenin signalling pathway promotes germ cell proliferation. Then, in addition to the retinoic acid (RA) signaling pathway, RSPO1 contributes in meiosis entry as a parallel pathway (indicated by the presence of two parallel arrows). RSPO1 promotes *Stra8* expression and subsequently meiosis initiation. Although we showed that RSPO1 is a direct activator of β-catenin in XX germ cells suggesting that RSPO1 is also acting via β-catenin in this developmental process, the direct inactivation of β-catenin in XX germ cells remains to be done. Yellow-filled circles: undifferentiated germ cells; blue-filled and pink-filled circles: respectively male and female germ cells at the differentiating stage. RA: Retinoic Acid.

RSPO1 is an activator of β-catenin both in germ cells and somatic cells and thus it is difficult to uncouple the germinal and somatic effect of RSPO1. However, depletion of β-catenin signaling in the *Sf1* positive somatic cell lineage did not affect either germ cell proliferation or meiosis in XX *Catnb^flox/flox^*; *Sf1:Cre^Tr^* ovaries (our data and [63]). This suggests that the germ cell defects observed in XX *Rspo1^−/−^* gonads are rather due to impairment of β-catenin signaling within germ cells. We cannot exclude that the germ cell proliferation defect, impaired entry into meiosis and germ cell sex reversal in XX *Rspo1^−/−^* mutants are an indirect consequence of loss of *Rspo1*-mediated β-catenin signalling in a *Sf1*-negative expressing cell type in the gonad. However, any indirect effect of *Rspo1* deficiency on XX germ cells will have to occur in the absence of any detectable Sertoli cell differentiation. Given that β-catenin signalling is active in female germ cells in a sex-specific and *Rspo1*-dependent manner, and that these germ cell phenotypes are observed prior to detectable Sertoli cell differentiation, and do not occur in XX *Catnb^flox/flox^*; *Sf1:Cre^Tr^* gonads, we favour the interpretation that loss of *Rspo1*-mediated β-catenin signalling in the germ cells themselves is responsible for the germ cell proliferation defect, impaired entry into meiosis and germ cell sex reversal in XX *Rspo1^−/−^* mutants. At present, how β-catenin promotes *Stra8* expression and represses *Nanos2* in XX germ cells remains to be elucidated (See Model in Fig. 9).

Whereas germ cell sex reversal is explained by regulation of key genes like *Stra8* and *Nanos2* in XX germ cells, it is surprising that Sertoli cell differentiation occurs so late, i.e. after sex reversal of germ cells in XX *Rspo1* mutant gonads. One possible explanation is that the differentiation of gonocytes in XX *Rspo1^−/−^* gonads influences masculinisation of the surrounding somatic cells promoting them to differentiate into Sertoli cells. In support of this hypothesis, germ cells can have a masculinizing effect on gonad development in some mouse models for sex-reversal [56], [70]. It would be interesting to determine whether signaling molecules such as prostaglandin D2 play a role in masculinisation of the somatic cells in XX *Rspo1^−/−^* gonads [56].

It is now clear that somatic sex determination is regulated by a tight balance between two different pathways [33], the SRY/SOX9/FGF9 pathway for testis development and RSPO1/WNT/β-catenin for ovarian differentiation [71]. Our data suggest that a similar balance exists for the sexual differentiation of germ cells and we have shown that RSPO1/β-catenin signaling has a key function in oogonial differentiation by regulating the proliferation and meiosis initiation of the XX germ cells.

## Materials and Methods

### Mouse strains and genotyping

The experiments here described were carried out in compliance with the relevant institutional and French animal welfare laws, guidelines and policies. These experiments have been approved by the French ethics committee Comité Institutionnel d'Ethique Pour l'Animal de Laboratoire (number:NCE/2011-12). All mouse lines were kept on a mixed 129/C57BL6/J background (4 to 5 back-crosses on C57BL6/J background, a purer background results in unhealthy animals). *Rspo1^−/−^* and *Axin2^+/LacZ^* transgenic mice were previously described in [6], [52]. *TNAP:Cre^TR^* mice [59] were mated with mice carrying the β-catenin exon3 floxed allele (*Catnb^ex3/+^*) [58] to obtain *Catnb^ex3/+^*; *TNAP:Cre^TR^* embryos. *Sf1:Cre^TR^* mice (kind gift from Keith Parker) [62] were mated with mice carrying the β-catenin conditional knock out allele (*Catnb^flox/flox^*: a kind gift from Rolf Kemler, Max Planck Institute, Freiburg, Germany) [72] to obtain *Sf1:Cre^TR^*; *Catnb^flox/flox^* embryos. *Oct4-GFP* embryos were generated by breeding *Oct4-GFP* homozygous transgenic male mice (a kind gift from Jenny Nichols, CSCR, Cambridge, UK) with CD1 female mice. Embryonic samples were collected from timed matings (day of vaginal plug = E0.5). Genotyping was performed using DNA extracted from tail tip of embryos or ear biopsies of mice with published primers related to the strains. The presence of the Y chromosome (*Sry* PCR) was determined as described previously [5]. *Pa*x6 primer set 5′ GCAACAGGAAGGAGGGGGAGA 3′; 5′ CTTTCTCCAGAGCCTCAATCTG 3′ was included in each PCR reaction as an internal control.

### Germ cell isolation

#### For FACS sorting

Urogenital ridges (gonad and mesonephros) were dissected from E13.5 *Oct4-GFP* embryos, sorted into testes or ovaries, and trypsinised to single-cell suspensions. The cells were then resuspended in ice-cold PBS and sorted for GFP fluorescence using a BD FACSAria II cell sorter. GFP-positive cells (germ cells) and GFP-negative (somatic cells) were isolated for each sex.

#### For XGal/immunostaining

Urogenital ridges were dissected from XY and XX E12.5 *Axin2^+/LacZ^* gonads and germ cells were isolated according to [56]. Germ cells were allowed to adhere on a 12 mm cover glass pre-coated with a 0.1 mg/mL solution of poly-L-Ornithine (Sigma cat P4957) for 2 hours at RT. They were then processed for XGal staining according to [73]. After 12 hours of staining at 37°C, they were washed in PBS, fixed in 4% PFA for 10 minutes and then processed for DDX4/MVH immunostaining (see below).

### Histological analysis

This technique was performed as described previously [6]. Urogenital organs were dissected, fixed in Bouin's solution overnight, and processed to obtain 5 mm or 3 mm thickness paraffin sections. For each genotype, 5 sections of 3 different embryos were processed for Haematoxylin and Eosin staining and quiescent germ cells were analysed with a light microscope, using a 100× objective. The quiescent state was identified by a uniform size of the gonocytes and the absence of any heterochromatin in the nuclei whereas the proliferative state was characterized by the variation in nuclear size, perinuclear heterochromatin (late S phase and G2 phase), and appearance of chromosome condensation and chromosomal threads (mitosis). In the present study, in the counts it was determined whether or not the cells were in one of the phases of a mitotic division. All the sections obtained from 2 XY wild type gonads and 6 XY *Catnb^ex3/+^*; *TNAP:Cre^Tr^* gonads were used for the quantification. Pictures were taken with an Axiocam mrm camera (Zeiss) and processed with Adobe Photoshop.

### X-gal staining and immunological analyses

These techniques were performed as described previously [6]. Embryonic samples were fixed with 4% paraformaldehyde overnight and then processed for paraffin embedding. Embryonic samples for cryosection were successively fixed 2 hours in 4% paraformaldehyde, washed in cold PBS, equilibrated in 10% sucrose during 3 hours, then in 30% sucrose overnight at 4°C, embedded in Cryomount (Histolab) and stocked at −80°C before cryosection. For each genotype, 5 cryostat or microtome sections of 8 µm thickness of 2 to 3 different embryos were processed for XGal staining or/and immunostaining. The following dilutions of primary antibodies were used: DDX4/MVH (cat 13840, Abcam), 1∶200, γH2AX (cat 16-193, Upstate) 1∶300, SCP3 (ab15091, Abcam) 1∶50, SOX9 (kindly provided by Michael Wegner) 1∶1500, active β-catenin (anti-ABC, clone 8E7, cat 05-665, Millipore) 1∶200, FOXL2 (ab5096, Abcam), 1∶250 and SDMG1 (generated in [32]) 1∶2000. DAPI (blue) was used to detect nuclei. For histology, 5 µm thickness sections of 2 embryos of each genotype were stained with Hematoxylin & Eosin. Fluorescent studies were performed with a motorized Axio ImagerZ1 microscope (Zeiss), and pictures were taken with an Axiocam mrm camera (Zeiss) and processed with Adobe Photoshop.

### Counting of proliferating, apoptotic or meiotic germ cells

Urogenital organs were dissected and fixed in 4% paraformaldehyde, and processed to obtain 5 µm paraffin sections as described above.

#### Counting of meiotic germ cells

For each genotype, 5 sections of 3 different embryos were processed for immunohistological experiments with DDX4/MVH antibody, and SCP3 and γH2AX at respectively E14.5 and E16.5. Then the percentage of meiotic germ cells versus total germ cells (MVH positive) was determined. Both γH2AX and MVH-positive germ cells were quantified on the same sections after a co-immunostaining, whereas SCP3-positive and MVH-positive germ cells were independently quantified on consecutive sections since a co-immunostaining was not technically possible.

#### Counting of proliferating and apoptotic germ cells

For each genotype, 8 sections of 3 embryos (24 pictures per genotype) were processed for immunohistological experiments with DDX4/MVH antibody. Then proliferation analysis was performed on the same sections by way of 5-Bromo-2′-deoxy-Uridine labelling and detection using an appropriate kit (cat 11 296 736 001, Roche) at E12.5. TUNEL analysis was performed with the *In Situ* Cell Death Detection kit, TMR red (cat 11 684 795 910, Roche) at E14.5 and E16.5. Total germ cells, proliferating (both BrdU- and MVH-positive cells) and apoptotic (both TUNEL- and MVH- positive cells) were quantified on the entire section using ImageJ software with a macro designed by Cédric Matthews (plateform of microscopy, IBDC Nice). For each picture, the number of BrdU-positive/TUNEL-positive germ cells and the number of total germ cells (MVH positive) were counted. Then the percentage of BrdU-positive/TUNEL-positive germ cells versus total germ cells (MVH positive) was determined. For each genotype (∼24 pictures), the mean and mean+1 SEM of these percentages were calculated and reported on a graph after statistical analysis (for details, see paragraph Statistical Analysis above).

### In situ hybridization

In situ hybridization was carried out as described previously [6]. *Stra8*, *Cyp26b1*, *Oct4* and *Fgf9* riboprobes were a kind gift of David Page, Peter Koopman, and Jennifer Colvin respectively. Digoxigenin riboprobe for *Nanos2* was generated by amplifying a cDNA fragment by RT-PCR from *Nanos2* (NM_194064, bases 126–696) and inserting it into TA cloning vector pCR2.1- TOPO (Invitrogen). The plasmid was then linearized by BamHI and transcribed with T7 RNA polymerase in the presence of Dig-labeling mix (Roche).

### Quantitative PCR analysis

Individual gonads without mesonephros were dissected in PBS from E12.5 and E14.5 embryos and immediately frozen at −80°C. RNA was extracted using the RNeasy Qiagen kit, and reverse transcribed using the RNA RT–PCR kit (Stratagene). Primers and probes were designed by the Roche Assay design center (https://www.rocheappliedscience.com/sis/rtpcr/upl/adc.jsp). Primers: *Hprt1*: 5′-tcctcctcagaccgctttt-3′ and 5′- cctggttcatcatcgctaatc-3′ (probe 95), *Mvh*: 5′- ccaagatcaggggacacagt-3′ and 5′- ctttggtaagtgtcaccattgc-3′ (probe 77), *Nanos2*: 5′-aggtcccccgatctcaac-3′ and 5′-cagcatttcccagtgtttcag-3′ (probe 98), *Dnmt3L*: 5′-aaccgacggagcattgaa-3′ and 5′- ccgagtgtacacctggagagt-3′ (probe 34), *Tdrd5*: 5′- aggacaagcagagaggatgg-3′ and 5- tgacccctgttgtttcattcta-3′ (probe 102), *RARα*: 5′- agacacgcagacgggttg-3′ and 5′- gaggatgccactcccaga-3′ (probe 83), *RARβ*: 5′- caaacgaagcagggcttg-3′ and 5′- caccggcatactgctcaa-3′ (probe 63), *Axin2*: 5′- gcaggagcctcacccttc-3′ and 5′- tgccagtttctttggctctt-3′ (probe 50), *Lef1*: 5′- tgcctacatctgaaacatggtg-3′ and 5′- caagcttccatctccagaagag-3′ (probe 12), *LRP6*: 5′- tcctcgagctctggcact-3′ and 5′- cctccccactcagtccaata-3′ (probe 18), *Sf1*: 5′- cgtaaactgcgcacaggag-3′ and 5′- gtagattggttcaggggaagg-3′ (probe 105), *Bmp2*: 5′-cggactgcggtctcctaa-3′and 5′-ggggaagcagcaacactaga-3′ (probe 49), *Fst*: 5′-aagcattctggatcttgcaact-3′ and 5′-aggaaagctgtagtcctggtctt-3′ (probe 101), *Rspo1*: 5′- cgacatgaacaaatgcatca-3′ and 5′- ctcctgacacttggtgcaga-3′ (probe 5), *Stra8*: 5′- tgacgtggcaagtttcctg-3′ and 5′- gttgcaggtggcaaacatag-3′ (probe 107). For *Cyp26b1*, *Sox9*, *Fgf9* and *Pdgs* primers, see [6]. All real-time PCR assays were carried out using the LC-Faststart DNA Master kit Roche. QPCR was performed on cDNA from one gonad and compared to a standard curve. QPCR were repeated at least twice. Relative expression levels of each sample were determined in the same run and normalized by measuring the amount of *Hprt1* (total gonadal cells) or *Mvh* (germ cells) cDNA. For XX and XY control, n = 6, XX *Rspo1^−/−^*, n = 10 gonads. The results were analyzed using Sigma Plot and Graphpad for statistical relevance. Graphs of QPCR results show mean ± SEM, * indicates p<0.05, ** indicates p<0.01 and *** indicates p<0.001.

### Statistical Analysis

Asterisks highlight the pertinent comparisons and indicate levels of significance: * indicates p<0.05, ** indicates p<0.01 and *** indicates p<0.001. Statistical significance was assessed using one-way ANOVA followed by Tukey-Kramer post test for selected pairs of genotypes. Graphs of QPCR results show mean, and mean+1 SEM.

## Supporting Information

Figure S1 **Control experiments for the purification of FACS sorted cells.** Quantitative RT-PCR analysis of *Mvh*, *Bmp2*, *Fst*, *Sf1 and Rspo1* expression in E13.5 germ cells (XX and XY *Oct4*-positive cells) and somatic cells (XX and XY *Oct4*-negative cells), using *Hprt* as the normalization control. Bars represent mean+1 SEM, n = 3 individual experiments.
(TIF)

Figure S2 **β-catenin signaling pathway is activated postnatally in XY proliferating germ cells.** Upper panel: X-Gal staining (AXIN2) and immunostaining with MVH (germ cells) or SDMG1 (Sertoli cells) in XY and XX *Axin2^+/LacZ^* gonads at P0 and P12. Black arrowheads: germ cells. Lower panel: *Rspo1 in situ* hybridization at P0 in XY gonads and epididymis (positive control, inset).
(TIF)
