# Supplementary figures and images for: RSPO1/β-Catenin Signaling Pathway Regulates Oogonia Differentiation and Entry into Meiosis in the Mouse Fetal Ovary
Source: PLoS One. 2011 Oct 3;6(10):e25641. doi: 10.1371/journal.pone.0025641 (PMC3185015; doi:10.1371/journal.pone.0025641)

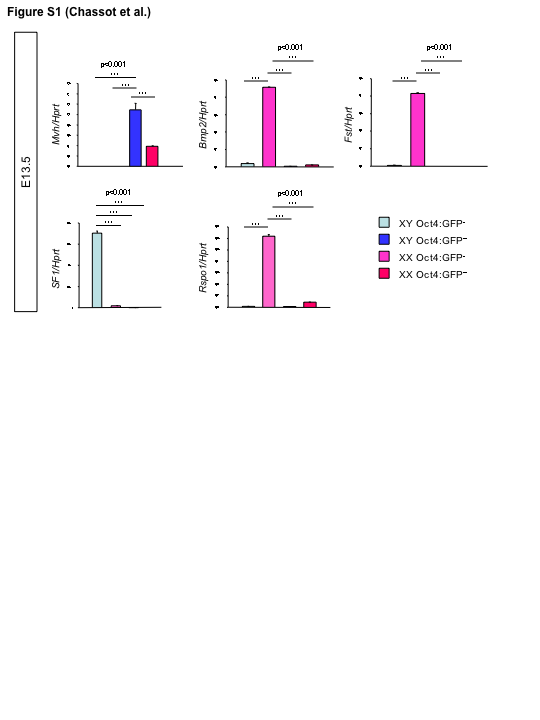

Supplement: Figure S1 — Control experiments for the purification of FACS sorted cells. Quantitative RT-PCR analysis of Mvh, Bmp2, Fst, Sf1 and Rspo1 expression in E13.5 germ cells (XX and XY Oct4-positive cells) and somatic cells (XX and XY Oct4-negative cells), using Hprt as the normalization control. Bars represent mean+1 SEM, n = 3 individual experiments. (TIF) [file pone.0025641.s001.tif]

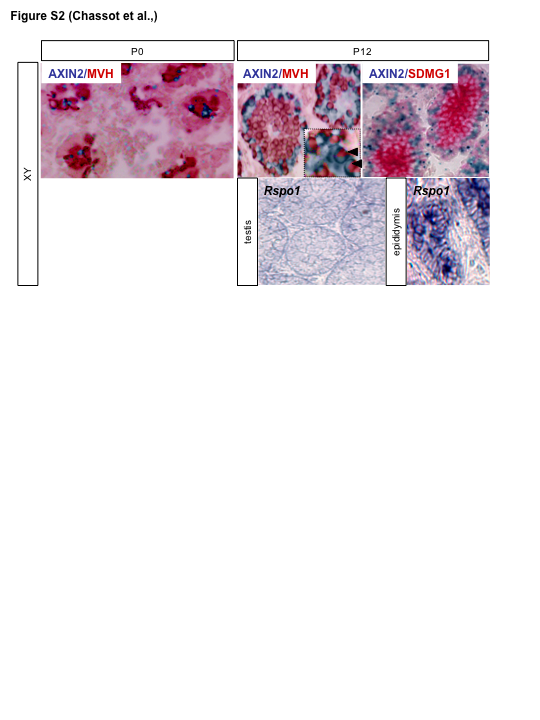

Supplement: Figure S2 — β-catenin signaling pathway is activated postnatally in XY proliferating germ cells. Upper panel: X-Gal staining (AXIN2) and immunostaining with MVH (germ cells) or SDMG1 (Sertoli cells) in XY and XX Axin2+/LacZ gonads at P0 and P12. Black arrowheads: germ cells. Lower panel: Rspo1 in situ hybridization at P0 in XY gonads and epididymis (positive control, inset). (TIF) [file pone.0025641.s002.tif]
